# Nutritional composition and antinutrient content of *Heteromorpha arborescens* (Spreng.) Cham. & Schltdl. leaves: An underutilized wild vegetable

**DOI:** 10.1002/fsn3.1978

**Published:** 2020-12-24

**Authors:** Taiwo Oluwafunmilola Abifarin, Gloria Aderonke Otunola, Anthony Jide Afolayan

**Affiliations:** ^1^ Medicinal Plants and Economic Development (MPED) Research Centre, Department of Botany, Faculty of Science & Agriculture University of Fort Hare Alice South Africa

**Keywords:** antinutrients, *Heteromorpha arborescens*, malnutrition, mineral, proximate, vitamins

## Abstract

The nutritional and antinutrient composition of *Heteromorpha arborescens* (Spreng.) Cham. & Schltdl. leaves was reported in this study. Proximate analysis revealed the presence of 8.5% total ash, 4.92% crude fat, 8.41% moisture, 15.74% crude protein, 21.48% crude fiber, 40.95% carbohydrates, and 271.04 kcal/100 g energy value. Mineral analysis showed that *H. arborescens* leaves are very rich in K, Ca, and Fe. Considerable amounts of Mg, Mn, Na, P, Cu, and Zn were also present. Vitamin analysis showed that the plant has a high content of vitamins A, C, and E. The antinutrients evaluated were phytate, oxalate, saponin, and alkaloids, all of which were below toxic levels except for saponin which was observed at moderately high level. The results credibly indicate that *H. arborescens* leaves are nutrient‐rich and can contribute effectively to the daily nutrient requirements alongside its therapeutic properties.

## Graphical abstract

Proximate contents of *H. arborescens* leaves were present in significant amounts and very rich in K, Ca, and Fe with considerable amounts of Mg, Mn, Na, P, Cu, and Zn .Vitamins A, C, and E were observed in high amounts. Phytate, oxalate, saponin, and alkaloids were below toxic levels except for saponin which was in moderately high level. The results indicate that *H. arborescens* leaves are nutrient‐rich and can contribute effectively to the daily nutrient requirements alongside its therapeutic properties.

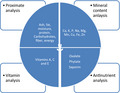

## 1 INTRODUCTION

There are many native medicinal plants which have been reported to be useful as vegetables, spices, and medicine (Okwu 2005; Muhammed et al., 2011). The World Health Organization has confirmed that above 68% of the global population depends mainly on medicinal plants to meet their health needs (WHO 2003), as most of them are nutrient‐rich, effective, safe, relatively cheaper, and readily available for the use of people especially the rural populace. Vegetables are very important sources of these nutrients which contribute markedly to food security and healthy diets for humans especially for children, lactating and pregnant women. (Nesamvuni et al., 2001; Yang & Keding, 2009). It is well known that besides providing the satisfaction of fullness, many plants are consumed because of their perceived medicinal needs. However, due to lack of knowledge about some of the medicinal food plants which are necessary to combat malnutrition and meet the required nutrient intake levels, such plants are underutilized. The study of plant foods with nutraceutical, pharmaceutical, nutritional, and functional properties is on the increase, and many plants with multi‐functional properties are now gaining recognition and usage (Otunola & Afolayan, 2019).

One of such underutilized multipurpose plants is *Heteromorpha arborescens* (Spreng.) Cham. & Schltdl. which belongs to the Apiaceae family. It is a large shrub‐like, medium deciduous tree widely distributed over tropical and temperate regions in Africa. The plant has gained great importance in parts of Africa especially in east and southern Africa because of its nutritional components, biological activities, and therapeutic efficiency (Maroyi, 2018). The leaves and roots are used for the treatment of inflammation, abdominal pains, and as general analgesic (Mc Graw et al. 1997; Lundgaard et al., 2008). Other uses are for intestinal deworming, jaundice, kidney problems, diabetes (Moshi and Mbwambo, 2002; Erasto et al., 2005), to treat mental disorders (Hutchings et al., 1996; Palmer & Pitman, 1972; Van Wyk and Oudtshoorn, 2013), fever, and malaria (Fowler, 2006; Lundgaard et al., 2008; Schmidt et al., 2002; Van Wyk and Oudtshoorn, 2013). As a result of its perceived great nutritional value, the leaves are eaten as vegetables in Kenya (Bussman 2006) and the roots are fed to malnourished children in Botswana and Swaziland (Setshogo and Mbereki, 2011). However, the leaves are not consumed as vegetable in some parts of Africa including South Africa possibly due to lack of information of its nutritional potentials. Furthermore, despite the nutritional and medicinal significance of *H. arborescens* leaves, to the best of our knowledge there is no documented report on the proximate, mineral, vitamins, and antinutritional compositions of *H. arborescens* leaves. Therefore, the present study is aimed at investigating the nutritional profile of *H. arborescens* leaves toward an informed validation of its traditional use as a vegetable, while encouraging its possible inclusion in human diets especially in South Africa.

## 2 MATERIALS AND METHODS

### 2.1 Collection and preparation of plant materials

Fresh leaves of *H. arborescens* were collected from a site located on latitude 32° 47′ 50.4″ S, 26° 52′ 41.8″ E along Hogsback road in Alice, Eastern Cape, South Africa, and it was authenticated by Prof. Cupido, a taxonomist at University of Fort Hare. The freshly collected leaves were washed, oven dried at 40°C, pulverized, and stored at 4°C until further analysis.

### 2.2 Proximate analysis

#### 2.2.1 Moisture content

An empty glass beaker was oven dried at 105°C for 1 hr, cooled in a desiccator, and weighed (*W*_1_). 2 g of the sample (*W*_2_) was weighed into the beaker, and both beaker and its content were oven dried at 105°C and cooled in a desiccator until constant weight was obtained (*W*_3_). The percentage moisture was calculated as:

$$\text{Moisture}\;\left(\%\right)=\frac{W_{2}‐\;W_{3}}{W_{2}‐\;W_{1}}\times 100.$$

#### 2.2.2 Ash content

The ash content was determined by as previously described (Unuofin et al., 2017). A crucible was washed, dried (105°C) for 1 hr, and left to cool in a desiccator, and the weight was measured (*W*_1_). Then, 2 g of the dried sample was measured into the weighed crucible and the new weight was measured (*W*_2_). The crucible with its content was then put into a muffle furnace for 1 hr at 250°C and again for another 5 hr (550°C) for complete ashing. The samples were left to cool in a desiccator, and the final weight was measured (*W*_3_). The percentage ash was calculated as:

$$\text{Ash content}\;\left(\%\right)=\frac{W_{2}‐\;W_{3}}{W_{2}‐\;W_{1}}\times 100.$$

#### 2.2.3 Crude fat

The crude fat content was determined by the AOAC (2016) method. Briefly, 5 g of the pulverized sample was extracted in 100 ml of diethyl ether for about 24 hr. The extract was filtered into a beaker of known weight (*W*_1_). It was thereafter made up to 100 ml with diethyl ether and shaken for another 6 hr; the filtrate was collected into *W*_1_. The ether was concentrated to dryness in a steam bath and oven dried at 40–60°C, and then the beaker was weighed again (*W*_2_). The crude fat content was estimated as:

$$\%\;\text{Crude fat}=\frac{W_{2}‐W_{1}}{\text{Original weight of sample}}$$

#### 2.2.4 Crude fibre content

Crude fibre of *H. arborescens* leaves was determined as described by Unuofin et al. (2017). Briefly, 2 g of the pulverized sample was treated with 100 ml of 1.25% H_2_SO_4,_ and the solution was boiled for 30 min and filtered under pressure, and the residue was washed with boiling water. This residue was further treated and boiled with 100 ml of 1.25% NaOH solution. The final residue was then dried at 100°C and cooled in a desiccator and weighed (*W*_1_). The final residue was thereafter subjected to heating in a muffle furnace at 550°C for 5 hr, transferred to cool in a desiccator, and reweighed (*W*_2_). The percentage crude fibre was calculated as:

$$\text{Crude}\;\text{fibre}\;\text{(\textbackslash{}\% )}=\frac{W_{2}‐W_{1}}{\text{Original weight of sample}}$$

#### 2.2.5 Carbohydrate content

The carbohydrate content was calculated as:

%Totalcarbohydrate=100‐(%moisture+%totalash+%crudefat+%crudefibre+%crudeprotein).

#### 2.2.6 Energy value

The energy value was determined by multiplying the values obtained for carbohydrate, crude fat, and crude protein by 4, 9, and 4 respectively and summing up the products. It was calculated as:

Energyvalue(kcal/100g)=(carbohydrate×4)+(crudefat×9)+(crudeprotein×4).

#### 2.2.7 Crude protein content

The Kjeldahl method described in the AOAC (2005) was used to determine the protein content. Exactly 2 g of the sample was weighed into a 250 ml digestion flask containing a mixture of 20 ml of concentrated H_2_SO_4_ and a digestion tablet. The mixture was boiled (until a clear/transparent residue was obtained), allowed to cool, diluted with 250 ml distilled water, transferred into a 500 ml Kjeldahl flask containing 50 ml of 40% NaOH solution, and thereafter, subjected to distillation. 150 ml of the distillate was then collected into a flask containing 100 ml of 0.1N HCl. This was then titrated against 2.0 mol/L NaOH with methyl orange as indicator. The end point was marked by a color change to yellow. The percentage nitrogen content was calculated as:

$$\frac{\left[(\text{ml}\;\text{standard}\;\text{acid}\times \text{N of acid})‐(\text{ml}\;\text{blank}\times N\;\text{of}\;\text{base})]‐(\text{ml}\;\text{std}\;\text{base}\times N\;\text{of}\;\text{base})\right]}{\text{Weight}\;\text{of}\;\text{sample}\;\text{(g)}}\times \;1.4007.$$

Where, *N* = normality, percentage crude protein was obtained by multiplying the nitrogen value by a constant value of 6.25. %; Crude protein = Nitrogen in sample × 6.25.

#### 2.2.8 Antinutrient content

##### Phytate

Phytate content was determined as previously described by Talabi et al. (2016). Briefly, 2 g of the sample was soaked in 100 ml of 2% HCL for 3 hr and filtered with Whatman no 1 filter paper. 25 ml of the filtrate was thereafter transferred into another conical flask and to it; 5 ml of 0.3% ammonium thiocyanate solution plus 53.3 ml of distilled water was added. The solution was titrated against standard iron III chloride solution (0.001 95 g of iron per mL) until a reddish brown color which persisted for 5 min was obtained. Phytate content was calculated as: Phytate (%) = Titer value × 0.001 95 × 1.19 × 100.

##### Oxalate

Oxalate content was determined as described by Ohikhena et al. (2017). Briefly, 1 g of sample was weighed into a conical flask containing 75 ml of 3 M H_2_SO_4_. The solution was properly mixed and filtered. 5 ml of the filtrate was heated to 90°C and then titrated against 0.05 M of KMnO_4 _until there was a color change which persisted for about 30 s. The oxalate content was calculated by taking 1 ml of 0.05 M of KMnO_4_ as equivalent to 2.2 mg oxalate.

#### 2.2.9 Alkaloid content

Alkaloid content of *H. arborescens* leaves was determined as previously described by Oyeyinka and Afolayan (2019). 0.5 g of the sample was mixed with 200 ml of 10% acetic acid in ethanol. The mixture was covered, incubated at room temperature for 4 hr, filtered, and concentrated to about a quarter of its original volume in a water bath. Thereafter, concentrated ammonium hydroxide was added drop wise to the extract till complete precipitation was attained. The solution was allowed to settle, and the precipitate formed was washed with dilute ammonium hydroxide and then filtered. The residue was oven dried at 40°C and weighed, and the alkaloid content was calculated as:

%Alkaloid=Finalweightofsample/Initialweightofsample×100.

#### 2.2.10 Saponin content

Saponin content of *H. arborescens* leaves was determined as previously described by Idris et al. (2019). 0.5 g of the sample was measured into a beaker containing 50 ml of 20% ethanol. The solution was heated in a hot water bath for 4 hr at 55°C and filtered, and the residue re‐extracted with another 50 ml of 20% ethanol. The filtrates were mixed together and concentrated to 20 ml over the hot water bath at 90°C. The solution obtained was transferred into a 250 ml separating funnel containing 20 ml of diethyl ether. The aqueous layer was collected; 20 ml of n‐butanol was added to it and then washed thrice with 10 ml of 5% sodium chloride while the ether layer was thrown away. The mixture was oven dried (40°C) to constant weight, and the percentage saponin content of the sample was calculated as:

$$\%\;\text{Saponin}=\frac{\text{Weight}\;\text{of}\;\text{final}\;\text{filtrate}}{\text{Weight}\;\text{of}\;\text{sample}}\times 100.$$

### 2.3 Mineral analysis

Ca, Mg, K, P, Na, Zn, Mn, Cu, and Fe contents of *H. arborescens* leaves were quantitatively analyzed using Inductively Coupled Plasma‐Optical Emission Spectrometer (ICP‐OES; Varian 710–ES series, SMM Instruments, Cape Town, South Africa).

#### 2.3.1 Vitamin A (retinol)

Vitamin A content of the leaves was determined as described by Famewo et al. (2018). 1.0 g of the pulverized sample was soaked in 20 ml of petroleum ether for 1 hr. The mixture was filtered and evaporated to dryness, and 0.2 ml solution of chloroform‐acetic anhydride was then added to the residue. Thereafter, 2 ml of TCA‐chloroform (1:1 v/v) was added to the solution, and absorbance was measured at 620 nm using UV‐3000PC. The standard (Retinol) was prepared in the same manner with gradient concentration ranging from 0.2 mg/ml ‐ 1.0 mg/ml. The vitamin A content of the sample was determined as retinol equivalent in mg/g, from the standard curve from the equation: *Y* = 0.9178 x‐ 0.02, *R* ^2^ = .9979.

#### 2.3.2 Vitamin C (Ascorbic acid)

Vitamin C content of the sample was determined as described by Njoku et al. (2015). 1 g of the sample was soaked with 20 ml of 0.4% oxalic acid and filtered with Whatman No 1 filter paper. Thereafter, 9 ml of indophenol reagent was added to 1 ml of the filtrate, and the absorbance was measured at 520 nm. The vitamin C content of the sample was determined as retinol equivalent in mg/g, from the standard curve from the equation: *Y* = 2.7867x ‐ 0.6521, *R* ^2^ = .9384.

#### 2.3.3 Vitamin E (Tocopherol)

Vitamin E content of the sample was determined as previously described by Njoku et al. (2015). 1 g of the sample was soaked in 20 ml of ethanol and filtered. Thereafter, 1 ml of 0.2% ferric chloride in ethanol and 1 ml of 0.5% α ‐dipyridyl solution were added to 1 ml of the filtrate. The solution was further diluted with water to 5 ml, and the absorbance was measured at 520 nm. The vitamin E content of the sample was determined as retinol equivalent in mg/g, from the equation: *Y* = 0.501 x + 3.2723, *R* ^2^ = .9661.

## 3 RESULTS

### 3.1 Proximate composition

The proximal composition of *H. arborescens* leaves is presented in Table 1. The results showed that the leaves of the plant had considerable quantities of total ash, crude fat, moisture, crude protein, crude fiber, and carbohydrate contents with very high estimated energy value.

**Table 1:** Proximate composition of *Heteromorpha arborescens* leaves

Proximal parameters	Composition (%)
Total ash	8.5 ± 0.15
Crude fat	4.92 ± 0.1
Moisture	8.41 ± 1.10
Crude protein	15.74 ± 1.26
Carbohydrates	40.95 ± 0.03
Energy (Kcal/100 g)	271.04 ± 6.13
Crude fiber	21.48 ± 1.87

Note

Values are expressed as mean ± *SD*, *n* = 2.

### 3.2 Mineral composition

Nine minerals (Ca, Mg, K, P, Mg, Na, Zn, Mn, Cu, and Fe) from *H. arborescens* leaves were analyzed (Table 2).

**Table 2:** Mineral composition of *Heteromorpha arborescens* leaves

Minerals	Composition (mg/100 g)
Calcium	1565 ± 0.03
Magnesium	400 ± 0.01
Potassium	1685 ± 0.02
Phosphorus	55 ± 0.00
Sodium	75 ± 0.00
Zinc	2.1 ± 0.03
Manganese	9.55 ± 0.05
Copper	0.75 ± 0.02
Iron	12.55 ± 1.15

Note

Values are expressed as mean ± *SD*, *n* = 2

### 3.3 Antinutrient composition

**Figure 1:**
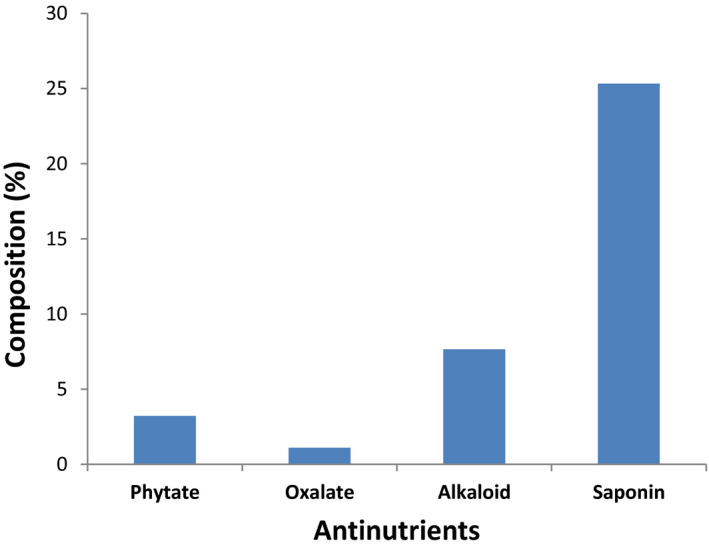
Antinutrient compositions of *Heteromorpha arborescens* leaves

The antinutritional composition of *H. arborescens* leaves is presented in Figure 1. Phytate, oxalate, saponin, and alkaloid contents were present as (3.22 ± 0.02) %, (1.1 ± 0.22) %, (25.33 ± 2.3) %, and (7.65 ± 0.45) %, respectively.

### 3.4 Vitamins A, C, and E compositions

**Figure 2:**
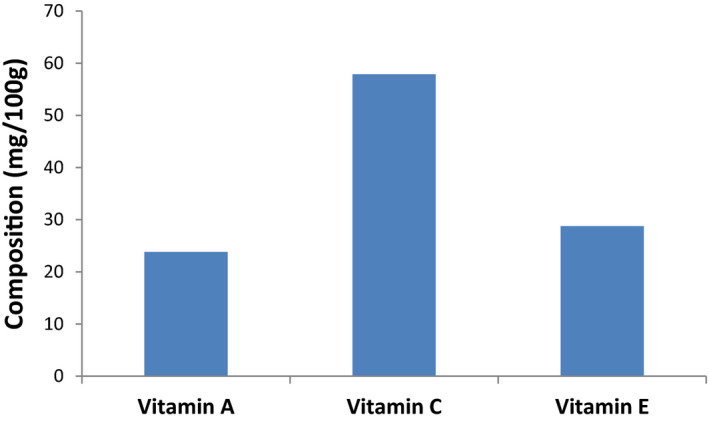
Vitamin composition of *Heteromorpha arborescens* leaves

Vitamin analyses of *H. arborescens* leaves (Figure 2) revealed the presence of Vitamins A (23.82 mg/100 g) and E (28.77 mg/100 g) was observed in remarkable quantities. However, vitamin C (57.89 mg/100 g) content was the highest.

## 4 DISCUSSION

Plants are rich sources of nutrients, and there has been an increasing global interest in the investigation of the nutritional and antinutritional compositions of medicinal food plants in order to tackle malnutrition, increase food security, and prevent diseases all over the world.

Protein is crucial for several body functions such as production of hormones, enzymes, maintenance of fluid balance, boosting the immune system, and body building (Emebu and Anyika, 2011; Achi et al., 2017). The protein content of *H. arborescens* leaves was observed to be moderate, and it can therefore be considered a good source of protein and potential protein supplements in the diet. Carbohydrate content indicates the energy content, and it is efficient for oxidation of fats (Omoyeni & Adeyeye, 2009). Considering the substantial quantity of carbohydrate observed in the plant, it can be regarded as a good dietary energy source. Dietary fiber functions in the regulation of bowel movement, proper digestion, and effective eradication of wastes from the body. They also lower the serum cholesterol; the risks of coronary heart diseases, hypertension, constipation, diabetes, colon, and breast cancers (Narzary & Basumatary, 2019; Viuda‐Martos et al., 2010). The high fiber content exhibited by *H. arborescens* leaves is indicative of its great health benefitting potential. Vegetables are generally characterized by low lipid contents (Achi et al., 2017); therefore, low fat content observed in *H. arborescens* leaves supports this fact and suggests its potential to maintain body weight, for the management of obesity, cardiovascular, and other high fat associated diseases (Adegbaju et al., 2019; Otunola & Afolayan, 2019). The value of ash content is suggestive of its elemental composition. Considerable ash content observed in this study suggests the moderate elemental composition of *H. arborescens* leaves. Likewise, the low moisture content of the samples reveals its long shelf‐life and low risk of microbial contamination, since high moisture content could increase microbial action that can lead to spoilage (Olumide et al., 2019; Ooi et al., 2012). The high energy content shown is attributed to the high carbohydrate content, which suggests that it can be a good source of dietary energy.

The results of the present study are comparable to other findings on proximate compositions of selected vegetables belonging to the Apiaceae family; although the fiber content of *H. arborescens* leaves was far higher than values obtained for *Coriandrum sativum* and *Daucus catota* L. According to Ayeni et al. (2018), the proximate analysis on *Daucus catota* L. revealed the presence of moisture (10.23%), ash (12.99%), crude protein (11.75%), carbohydrate (51.81%), energy (358.93%), crude lipid (10.37%), and fiber contents (9.07%). Similarly, Javid et al. (2009) and Ghajarieh Sepanlou et al. (2019) investigated *Coriandrum sativum* and *Eryngium caeruleum* respectively and observed the presence of moisture (6.65%, 8.6%), ash (8.03%, 9.45%), crude protein (14.59%, 17.9%), carbohydrate (63.71%, 39.56%), energy (390.39%, 236.78%), crude lipid (9.83%, 1.54%), and fiber contents (5.53%, 22.63%) for *Coriandrum sativum* and *Eryngium caeruleum,* respectively.

Minerals such as potassium, calcium, iron sodium, magnesium, phosphorus, manganese, zinc, and copper are crucial for normal body development and maintenance (Haruna et al., 2015). They help in sustaining and improving the functions of the, muscles, heart, and brain as well as the production and maintenance of strong bones and teeth (Jequier and Constant 2010). Magnesium is required in the plasma and extracellular fluid to maintain osmotic equilibrium. It also functions in many biochemical reactions including oxidative phosphorylation, glycolysis, and protein synthesis (Gröber et al., 2015; Thomas et al., 2000). Phosphorus plays a vital role in energy generation, maintenance of bones, teeth and muscles, and cell growth and also provides the structural framework for DNA and RNA (Gharibzahedi & Jafari, 2017). It also functions in regulating blood sugar levels and contraction of the heart (Achi et al., 2017). Potassium is crucial in maintaining normal cell functions, regular muscle contraction, and regulation of blood pressure. Calcium is required for bone and muscle formation, synaptic nerve impulse transmission and blood coagulation (Ozan and Akbulut 2008). High intake of calcium is highly recommended especially for children and pregnant women (Insel et al. 2011). Zinc functions in normal body development, protein synthesis, and wound healing. It also forms an important part of many enzymes in the human body (Afolayan & Jimoh, 2009). However, excessive consumption of zinc is dangerous to human health (Ogundola et al., 2018). Sodium is involved in the regulation of acid–base balance, normal cell function, transport of metabolites, nerve impulse transmission, and maintaining blood pressure (Unuofin et al., 2017). Iron is essential for hemoglobin formation; energy metabolism, and oxygen transport (Gaeta and Hider 2005; WebMD, 2014).

Manganese is necessary for all biosynthetic processes and maintenance of nerve and muscle electrical potentials. It also functions in oxygen transport from lungs to cells and activation of enzymes reactions associated with the metabolism of carbohydrate, fat, and protein (Jacob et al., 2015). Copper is a vital trace mineral, which is required in several enzymatic reactions, good functioning of organs in the body, collagen synthesis, energy generation, and formation of hemoglobin (DiNicolantonio et al., 2018; Saupi et al., 2009).

Sodium, phosphorus, manganese, iron zinc, and copper were present in considerable amounts while the calcium, magnesium, and potassium contents were relatively high. From the result of mineral composition obtained in the present study, it infers that *H. arborescens* leaves can serve as a natural sources of these essential minerals and even as potassium and calcium supplements. The results for mineral analysis obtained in this study are also comparable to previous observations by Tuncturk and Ozgokce (2015).

Antinutrients can be harmful to human health and may pose negative effect by reducing protein digestibility and mineral bioavailability. Low levels of phytate, oxalate, and alkaloids were observed with moderately high level of saponin. However, the presence of moderate level of saponin should not pose a problem if properly processed since processing decreases the level of antinutrients to permissible levels (Jimoh et al., 2010; Ndidi et al., 2014).

Research has linked the consumption of vitamin‐rich vegetables to a reduction in the risk of cancers and cardiovascular diseases (Abdou Bouba et al., 2012). Vitamins A, C, and E are great sources of antioxidants and are also vital in proper functioning of the eyes, growth and development, immune function, and reproduction (Shemishere et al., 2018). The significant amount of vitamins A and E and the high vitamin C content of *H. arborescens* leaves suggest that it may be beneficial in the prevention of a wide range of diseases such as coronary heart disease, anemia, heart diseases, cancers, and other degenerative diseases (Mgbeje et al., 2019). The present results revealed higher vitamins A, C, and E contents compared with some other common vegetables in Africa such as *Ocimum gatissimun, Gongronema latifolium, Piper guineense*, *Vernonia amygdalina* (Mgbeje et al., 2019), and *Celosia argentea* (Adegbaju et al., 2019). Vitamin C content of *H. arborescens* leaves has also proven to be higher than *Spinacea oleracea*, *Brassica oleracea,* and *Allium sativum* (Bangash et al., 2011).

## 5 CONCLUSION

Plants are good sources nutrients which are required for healthy human diets. This study revealed that *H. arborescens* leaves are a good source of nutrients and mineral elements (with low antinutrient content) that are highly beneficial to human health. Considering the high amounts of protein, fiber, calcium, carbohydrate, and other nutrients observed in *H. arborescens* leaves, it can serve as a good nutrient source to human diets. This finding also encourages its possible inclusion as a vegetable to the diet in South Africa.

## 6 Funding statement

This research was financially supported by Govan Mbeki Research and Development Center, University of Fort Hare (C127).

## 7 COMPETING INTERESTS

No competing interests were disclosed.

## ETHICAL APPROVAL

Ethical approval was granted by the University of Fort Hare Animal and Plant Use Research Ethics Committee, South Africa with protocol number OTA011SABI01/19/E.
